# Supplementary material for: Aqueous Binary Mixtures of Stearic Acid and Its Hydroxylated Counterpart 12-Hydroxystearic Acid: Cascade of Morphological Transitions at Room Temperature
Source: Molecules. 2023 May 25;28(11):4336. doi: 10.3390/molecules28114336 (PMC10254635; doi:10.3390/molecules28114336)
Supplement: Supplementary file 1 [file molecules-28-04336-s001.zip › molecules-2389446-supplementary.pdf]

Supplementary Materials

Aqueous Binary Mixtures of Stearic Acid and Its Hydroxylated Counterpart 12-Hydroxystearic Acid: Cascade of Morphological Transitions at Room Temperature

Maëva Almeida [1,2,†], Daniel Dudzinski [2,†], Catherine Amiel [1], Jean-Michel Guigner [3], Sylvain Prévost [4], Clémence Le Coeur [1,2,*] and Fabrice Cousin [1,*]

[1] Institut Chimie et Materiaux Paris Est, Université Paris Est Créteil, CNRS, UMR 7182, 2 Rue Henri Dunant, 94320 Thiais, France; maeva.ferreira-almeida@u-pec.fr (M.A.); catherine.amiel-guenoun@cnrs.fr (C.A.)

[2] Laboratoire Léon Brillouin, Université Paris-Saclay, CEA-CNRS UMR 12 CEA Saclay, 91191 Gif sur Yvette, France; daniel.dudzinski@cea.fr

[3] Institut de Minéralogie, de Physique des Matériaux et de Cosmochimie (IMPMC)-IRD-MNHN, Sorbonne Université & CNRS, UMR 7590, CEDEX 05, 75252 Paris, France; jean-michel.guigner@sorbonne-universite.fr

[4] Institut Laue-Langevin—71 Avenue des Martyrs, CS 20156, CEDEX 9, 38042 Grenoble, France; prevost@ill.fr

* Correspondence: clemence.le-coeur@cnrs.fr (C.L.C.); fabrice.cousin@cea.fr (F.C.)

† These authors contributed equally to this work.

SANS data fitting

All spectra were fitted with a lamellar stack Caillé model used in SasView 5.0.4 (http://www.sasview.org/.)., that is a random lamellar sheet with a Caillé structure factor. The intensity scattered by objects in solution is described as a product of form factor P(q) by structure factor S(q), where a random distribution in solution are assumed. In this model de Caillé S(q) is used for the lamellar stacks. Through the form factor it is possible to describe some specific characteristics of the spectrum, namely the thickness of the membrane δ, with the function $P(q) = \frac{2\Delta\rho^2}{q^2}(1 - cosq\delta)$.

We fixed the scattering length density of the solvent and the membranes (i.e. the fatty acids) and the total concentration, the others parameters were fitted and are present in the Table 1.

	Fit 1				Fit 2			
**Ratio**	**N_{layers} 1**	**Thickness (Å) 1**	***d*-spacing (Å) 1**	**Caillé parameter 1**	**N_{layers} 2**	**Thickness (Å) 2**	***d*-Spacing (Å) 2**	**Caillé parameter 2**
**0**	4	24.0	575	0.180	6	24.1	583	0.100
**0.05**	4	23.9	364	0.0354	4	24.0	371	0.0325
**0.1**	4	17.2	338	0.0334	5	24.4	337	0.0301
**0.15**	4	20.0	298	0.0255	6	23.5	304	0.0329
**0.25**	4	20.5	256	0.0349	7	24.2	256	0.0429
**0.4**	4	24.4	200	0.227	6	23.6	203	0.248
**0.5**	4	18.7	269	0.161	6	24.5	265	0.203
**0.6**	4	17.6	247	0.159	3	24.1	244	0.196
**0.75**	4	17.4	240	0.201	3	24.4	230	0.190
**0.9**	4	17.5	229	0.174	3	23.6	225	0.158
**1**	4	24.3	555	0.280	4	22.5	241	0.113

Table S1. Values of the parameters of the lamella obtained by fitting the intermediate and large q parts of the SANS data of Figure 5 (main text).

A low q the scattering curves for R=1, R=0.9 and R=0.75 were fitted with a model of core shell cylinder, with respective values of 202 nm, 210 nm and 250 nm for the radius, a constant thickness of 81 nm and a length of 20000 nm. The q-range probes experimentally does not allow to probe such a length. It was thus fixed arbitrary to this value according to the confocal microscopy data.

SANS data : Iq^{α} *versus q* representations

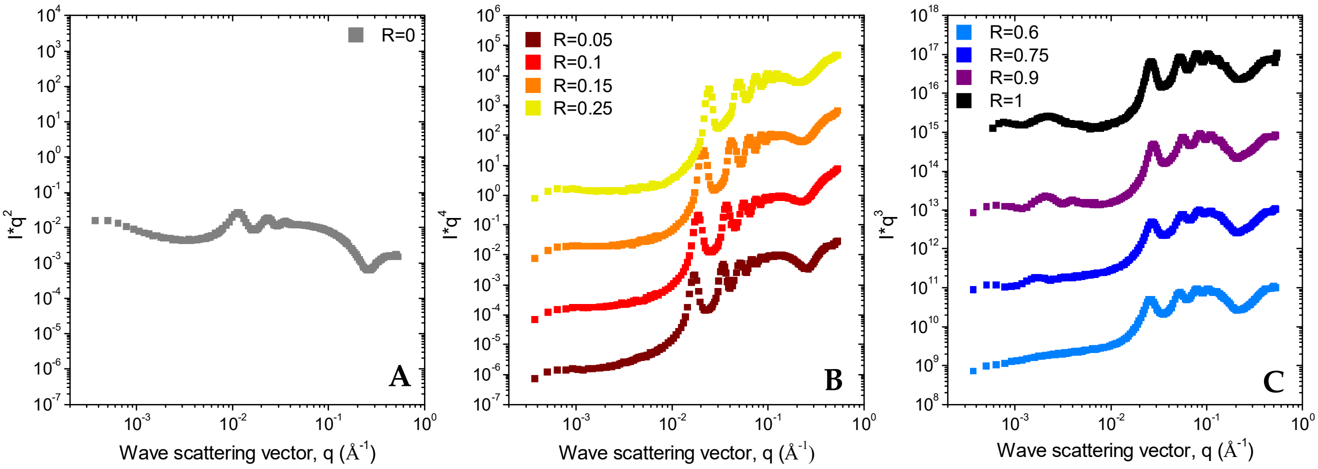

Figure S1 – (A) SANS data of R=0 represented as I*q^2 in function of wave scattering vector q. (B) SANS data of R=0.05, R=0.1, R=0.15 and R=0.25 represented as I*q^4 in function of wave scattering vector q. (C) SANS data of R=0.6, R=0.75, R=0.9 and R=1 represented as I*q^3 in function of wave scattering vector q.

Rheology

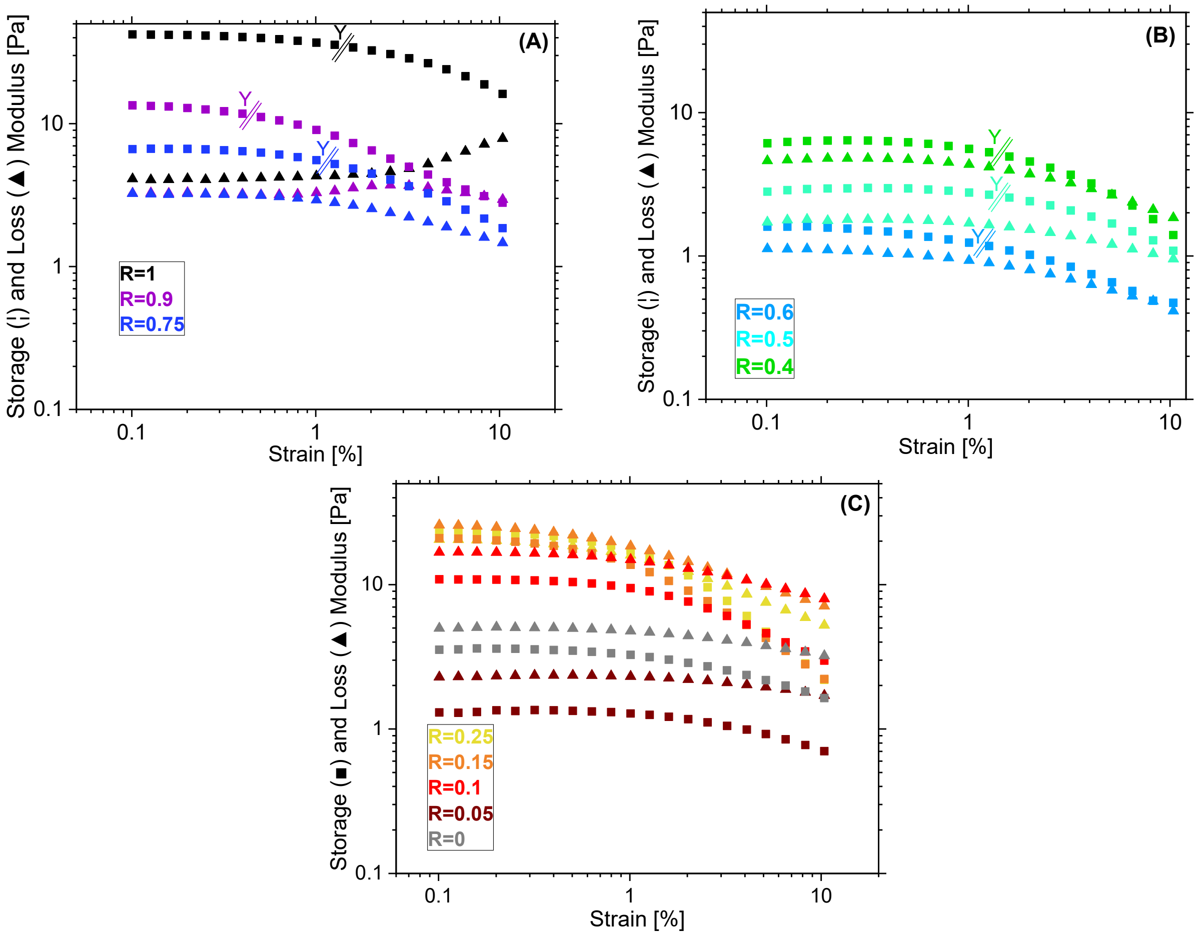

Figure S2a - Storage (■) and Loss (▲) Modulus as a function of the strain, at 1Hz, for R= 1 to 0.75 (A), 0.6 to 0.4 (B) and 0.25 to 0 (C). (The mark Y// point toward the yield strain determined through the asymptotic behavior of the stress vs strain curves).

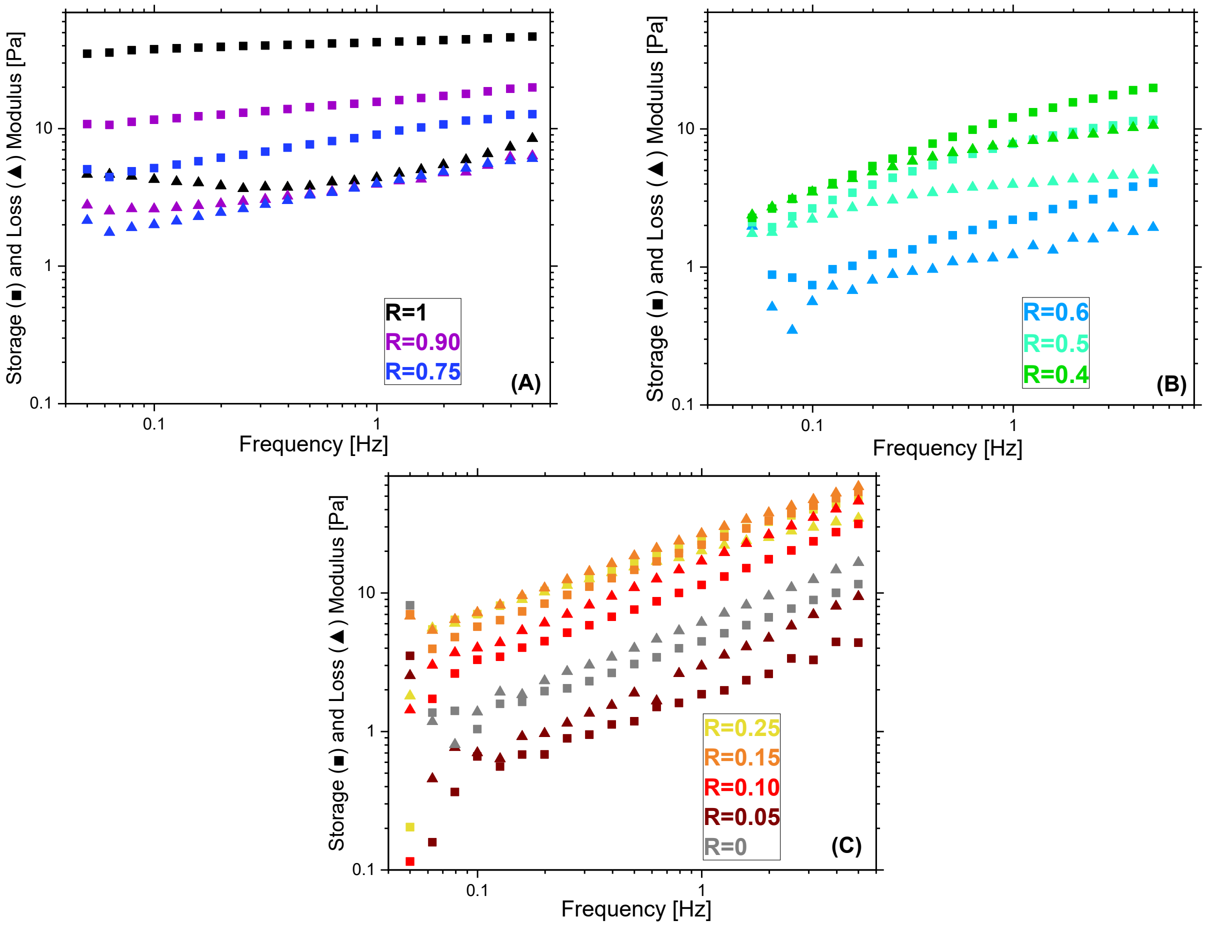

Figure S2b - Storage (■) and Loss (▲) Modulus as a function of the frequency, at 0.1% strain, for R= 1 to 0.75 (A), 0.6 to 0.4 (B) and 0.25 to 0 (C).

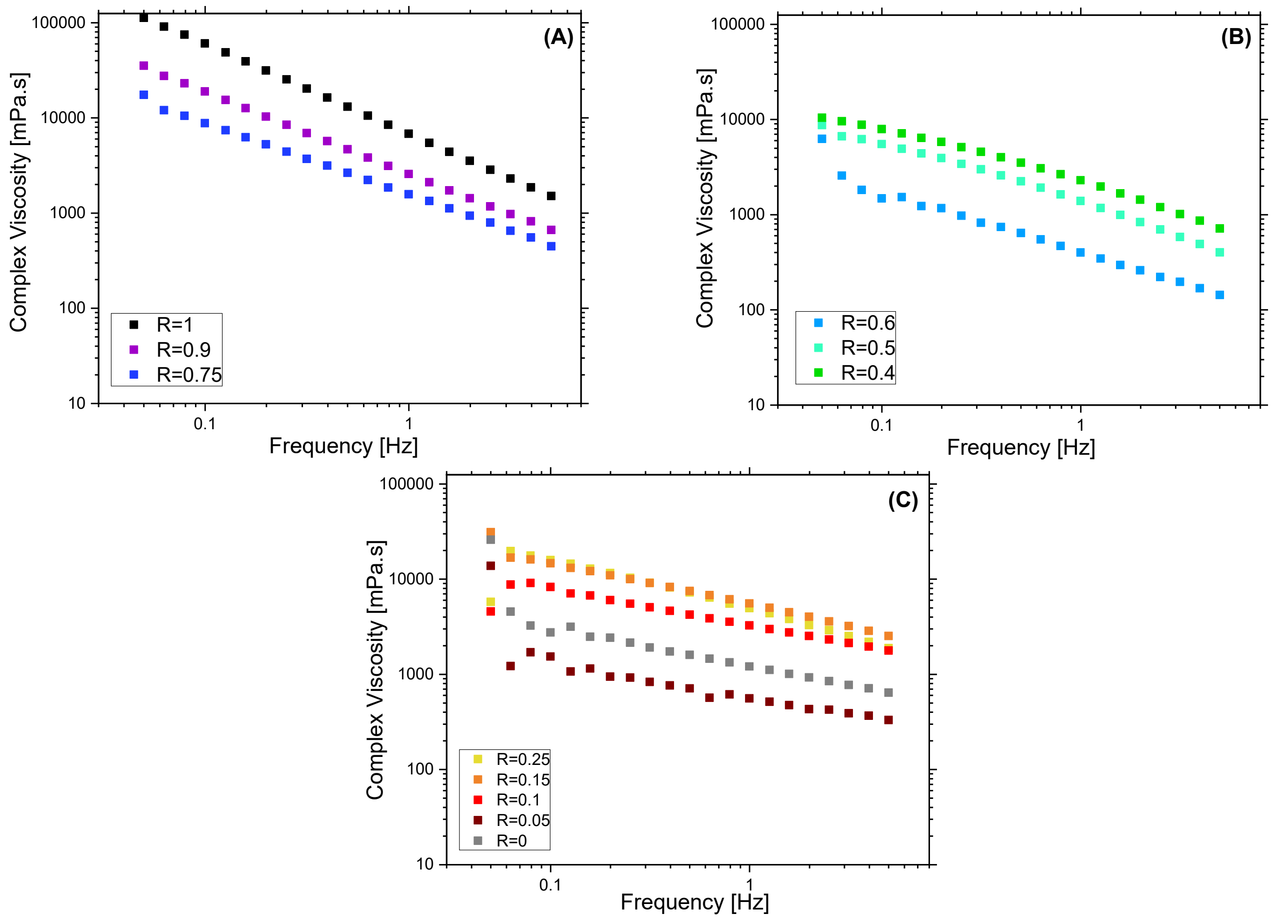

Figure S2c - Complex viscosity as a function of the frequency, at 0.1% strain, for R = 1 to 0.75 (A), R = 0.6 to 0.4 (B) and R = 0.25 to 0 (C).
